# What are we missing? Reflections on the ‘problem’ of missed appointments in the UK

**DOI:** 10.3310/nihropenres.14239.2

**Published:** 2026-05-19

**Authors:** Calum Lindsay, David A Ellis, David Baruffati, Mhairi Mackenzie, Catherine A O'Donnell, Sharon A Simpson, Geoff Wong, Andrea E Williamson

**Affiliations:** 1 General Practice and Primary Care, University of Glasgow College of Medical Veterinary and Life Sciences, Clarice Pears Building, 90 Byres Road, Glasgow, G12 8TB, UK; 2 Centre for Healthcare Innovation and Improvement Information, Decisions and Operations, Centre for Business Organisations and Society (CBOS), University of Bath, Bath, England, UK; 3 Urban Studies, University of Glasgow School of Social and Political Sciences, 27 Bute Gardens, Scotland, G12 8RS, UK; 4 Public Health, University of Glasgow School of Health and Wellbeing, Clarice Pears Building, 90 Byres Road, Glasgow, G12 8RS, UK; 5 Nuffield Department of Primary Care Health Sciences, University of Oxford, Oxford, England, UK

**Keywords:** missed appointments, non-attendance, did not attend (DNA), no-shows, health inequalities, access inequalities

## Abstract

**Background:**

Tackling missed appointments has become a prominent part of conversations about the ‘recovery’ of the UK NHS from the COVID-19 pandemic. With long waiting lists, delays in access to primary care, and overburdened staff, it seems logical that efforts should be made to reduce the time lost to unused appointment slots. Yet care needs to be taken to reflect on how and why missed appointments have become an area of focus, and to be transparent about what this means for patient care and health inequalities. This article provides a critical perspective on the current mainstream approach to missed appointments in policy, research and practice.

**Methods:**

The study applies Bacchi’s “what is the problem represented to be” method of policy analysis to provide critical commentary on findings from a realist review of 253 documents; interviews with 61 ‘key informants’ whose personal and professional experiences relate to missingness; and a series of co-design workshops with a Stakeholder Advisory Group of 16 professionals and experts-by-experience. It also includes an informal analysis of national news media and NHS news pieces covering missed appointments since 2015.

**Results:**

Analysis identified a consistent approach to missed appointments across research, practice and media and policy statements. This approach focuses on the impacts of missed appointments for services; places responsibility on the shoulders of patients; and limits interventions to things that seek to alter their behaviours in pursuit of a service-wide reduction in missed appointments. The hegemony of this approach is sustained by research that has failed to adequately engage with the most marginalised patients; failed to explore causes in depth; and failed to include an inequalities lens in its approach to interventions. We propose an alternative viewpoint – applying a missingness lens – built from an alternative evidence base that combines critical literature review, qualitative interviews, and participatory methods with those experiencing missingness and with key professionals.

**Conclusions:**

Embedding an alternative perspective in practice and in research has significant potential as a non-stigmatising, inequalities-based and effective approach to understanding and addressing missed appointments.

## Plain Language summary

How we define the causes and impacts of missed appointments in healthcare influences how they are addressed. How they are addressed has consequences for for people’s health. This paper explores how politicians, the media, NHS managers and researchers understand missed appointments. It argues that current approaches are based on flawed evidence, a narrow understanding of the issue, and use interventions that are likely to worsen health inequalities. They see missed appointments as a problem of waste for the struggling NHS, caused by irresponsible, misbehaving patients who must be punished or coerced to attend. This approach is now starting to influence Artificial Intelligence-based interventions that promise to predict who will miss appointments, saving even more money. Meanwhile, deeper causes are ignored because researchers have not carried out in-depth research with some patient groups, or developed different interventions to address access inequalities. Our paper describes the dangers of the current approach and outlines an alternative perspective. This focuses on how missed appointments impact the health of those who are ‘missing’, and how they can be addressed in a better, fairer way.

## Introduction

The UK National Health Service (NHS), like many health services globally, could be characterised as being in a state of “polycrisis” - beset by multiple, interlinked and mutually escalating ruptures that have significantly increased demand for care while restricting the system’s capacity to respond. ^2^ The idea that the NHS is facing crisis is not new, and crisis discourse reflects political concerns in defining the nature of crises and how they might be addressed. ^3^ While there is no doubting the scale of the challenges facing the health service, there is a need to closely investigate how those challenges are defined and how they are being addressed. This article explores how the ‘problem’ of missed appointments has become a prominent part of recent crisis conversations. The current crisis discourse - focused on waste, inefficiency, and the time and money lost to patients who do not attend – has not been closely examined, and the policy and practice emanating from it have not been subjected to critical scrutiny.

This piece is prompted by findings from a series of studies seeking to understand and address ‘missingness’: ‘the repeated tendency not to take up offers of care that has a negative impact on the person and their life chances’, evident in patterns of multiple missed appointments. ^4^ Prior epidemiological work covering 550,083 GP patient records in Scotland found that 19.0% of patients missed two or more GP appointments per year, and that these patients were more likely to have multiple, complex long-term health conditions, experience socioeconomic disadvantage, and have worse health outcomes including premature and all-cause mortality. ^5– 7^ These findings show that ‘missingness’ is a strong risk marker for negative outcomes, and the patterning of multiple missed appointments suggest that marginalised and excluded patient groups experience significant and enduring access barriers.

Very little research and policy space has been devoted to the specific experiences of ‘missing’ patients. To address this, our research team carried out a project combining realist review methods ^4^ with qualitative interviews ^8, 9^ and a process of co-produced intervention development ^10^ to explore the causes of missingness and potential solutions to address it. As this work proceeded, and particularly as stakeholder involvement progressed, we noticed a gulf between our developing understanding of the causes of missingness and much of the existing evidence, and thus significant differences between our proposed interventions and those proposed in research, policy, and practice. Throughout the study, we came across news articles, press releases, websites, even direct communications from our own GPs, that were negative, punitive and even hostile to patients who miss appointments – practices that stood in direct contradiction to the good practice suggested by interviewees, stakeholders, and the rare pieces of research that applied a different perspective (e.g. ^11, 12^).

While specific findings on causes of missingness and the ‘suite’ of interventions to address it are reported elsewhere, ^4, 8– 10^ this paper focuses on the discursive framing of the problem of missed appointments generally. Part research paper, part critical commentary, this paper uses Carol Bacchi’s “What is the problem represented to be” approach to policy analysis ^13, 14^ to provide a clear outline of the hegemonic ‘problem’ of missed appointments and to provide an alternative vision of the problem from the missingness work. It is our hope that this will support critical reflection from researchers, policymakers and practitioners.

## Methods

### Initial data collection

The Missingness project that informs this paper used three data sources. The first was a realist review of academic and grey literature on the causes of multiple missed appointments and existing intervention approaches, and the second a set of realist interviews with 61 people with personal or professional experiences relating to missingness. ^4, 8, 9^ Our third workstream convened several Stakeholder Advisory Group (StAG) workshops – one initial mini-StAG to guide theory development, and four further workshops throughout the project to support analysis and engage in intervention co-design. ^10^ The Group had 16 members (8 experts-by-experience and 8 professionals). All interview and StAG participants provided informed, voluntary consent to participation and to their anonymised data being used for publications. The College of Medical, Veterinary and Life Sciences Research Ethics Committee of the University of Glasgow gave ethical approval for the research (Reference: 200220187).

### Additional data collection

As the gulf between our growing understanding of missingness and the growing ‘crisis’ discourse became clear, we carried out a rapid, informal review of news materials covering missed appointments. Material was gathered through a search of national news outlets through the NEXIS news archive, the BBC News website, and the NHS News website. We searched NEXIS for article headlines featuring the terms: “missed appointments”, “non-attendance”, “no-show”, or “fail* to attend” and “NHS”, “health service”, “primary care”, “secondary care”, “elective”, “GP” or “general practice”. Articles were included if they were from national news outlets, if their headlines focused specifically on missed appointments, and if they were available. Searches of NHS and BBC News websites used similar terms (“no-show” OR “non-attendance“ OR “did not attend” OR “missed appointments”). Due to limitations with their search systems, results pages were listed by relevance and screened for headlines until they stopped returning relevant materials. This strategy was chosen in the absence of a fully articulated NHS policy about missed appointments, which are more often discussed in press releases or media statements. Materials were limited to those published since 2015. 62 articles from 10 sources are included. A full list of articles can be found in the supplementary materials. This set of articles is illustrative rather than comprehensive, and further media and policy analysis in this space would be beneficial.

### Patient and public involvement

PPI has been central to the missingness study. During the conceptualisation and development phase of the work, members of the research team consulted the Royal College of GPs Scotland Patient Participation in Practice (P3) and carried out pilot interviews, including interviews with people with lived experience of missingness. One Public Co-Investigator was involved in the funding application, with another contributing during the research to team meetings, recruitment and data collection, and analysis and dissemination. Our StAG members supported analysis of our realist review and refined our ‘programme theory’ of the causes of missingness, discussed and critiqued existing interventions, and co-designed a suite of interventions and principles to underpin them. ^10^

Their input provided a critical perspective on current discourse around missed appointments and contributed to development of the alternative ‘missingness lens’ reported below.

### Analysis: Bacchi’s ‘WPR’ approach

Although our project’s primary form of analysis was realist, concerned with identifying causal mechanisms and the contexts which interact to cause missingness, we also coded our data for constructions of the problem. Inspired by Bacchi’s ‘what is the problem represented to be’ approach to policy analysis, ^13, 14^ we created a framework for understanding how missed appointments are constructed, and how they might be understood differently. For Bacchi, policymakers construct ‘problematisations’ that contain presuppositions about the social world, and about how the people in it should be governed. ^14^ Policy analysis proceeds by asking six questions:

1. What’s the ‘problem’ represented to be in a specific policy or policy proposal?
2. What presuppositions or assumptions underpin this representation of the ‘problem’?
3. How has this representation of the ‘problem’ come about?
4. What is left unproblematic in this problem representation? Where are the silences? Can the ‘problem’ be thought about differently?
5. What effects are produced by this representation of the ‘problem’? ^14^
6. How/where has this representation of the ‘problem’ been produced, disseminated and defended? How has it been (or could it be) questioned, disrupted and replaced?

This analysis creates space to disrupt and displace authoritative approaches with alternatives, particularly those built from the perspectives of those targeted by policies but absent from their creation. ^15^ We applied this method first to research literature, interviews and stakeholder meetings, before refining it further through analysis of the media materials. This approach allowed us to outline two clear models of missed appointments: the hegemonic ‘classical’ approach; and the alternative ‘missingness lens’ (see Table 1). This commentary piece details these in depth to prompt critical reflection among policymakers, practitioners and researchers about their practice in relation to missed appointments.

**Table 1.** (Adapted from ^10^) showing the core tenets of the ‘classical’ model on the left, and of their equivalents when viewed using the ‘missingness lens.’

The classical model	A missingness lens
Patient ‘responsibilisation’	Services committed, resourced, incentivised to identify and address barriers.
Shallow, monocausal perspective	Complex causality for individuals, in wider contexts.
Technical, practical and logistical interventions	Relational responses focused on safety – structural, cultural, relational, psychological.
Standardised, service-wide interventions	Proportionate universalism and positive selectivism.
Biomedical models of healthcare	Condition competence, addressing social determinants of health, poverty and marginalisation.
Hierarchical, service-oriented solutions	A person-centred approach.

## Results

### The classical model

The dominant approach to missed appointments consists of several interlinked assertions:

1. Healthcare is delivered through appointments. Too many are missed - with concerns of an escalating problem, spiralling out of control.
2. Missed appointments are a problem *for* health providers facing a crisis of capacity and resourcing, and *for* other patients whose access is impacted.
3. Missed appointments are caused *by* the irresponsible, immoral, problematic behaviours of problematic patients; by universal (and understandable) practical/logistical issues; and by the inherent ‘vulnerability’ of some patients.
4. Interventions target patient behaviour to deliver benefits for health services by reducing the overall missed appointment rate.
5. If services can predict who will miss an appointment, they can target and optimise these interventions towards the most problematic – reducing waste and inefficiency further.

This basic problematisation is widespread. News articles, public statements and research papers often frame a significant, escalating problem, evidenced through references to rising numbers of missed appointments and rising costs – £1.2 billion across the NHS in 2024 ^16, 17^ with hundreds of millions lost in primary care alone. ^18^ In the crisis context, this is untenable and a cause for alarm. This money could be spent on other patients or other services; time lost could be used to see other people and reduce waiting lists. In a time of scarcity appointments are precious and valuable commodities in a way they may not have been before and wasting them is particularly egregious.

Health policy problematisations define who subjects are, what they might become, and how they should be governed biopolitically – through the regulation, administration and management of their lives and health, including through healthcare. ^14^ *Necro*political governmentality administers not life but death, determining who is worthy of care and who is “disposable”, ^19^ whose suffering is permissible and less worthy of attention because of their position in a moral hierarchy. ^20^ Mirroring wider discourse in health policy, ^21, 22^ statements about waste and lost capacity divide the patient population into those who are worthy of resources (and who pay for them through taxes), and those who are unworthy, whose difficulties are a function of their own shortcomings, and who drain resources from society. ^20, 23^

The classical model suggests that missing an appointment is a deviant behaviour with no deeper causes, reflecting a surface-level orientation to healthcare access issues. ^24^ This is visible in language which appears descriptive - ‘non-attendance’ or ‘did-not-attend (DNA)’ – as well as more evaluative phrases like ‘failed to attend’, ‘choosing’ not to attend, and in the directly exclusionary language of (ir) responsibility, entitlement, chaos, non-compliance. ^4, 12^ The NHS offered a free appointment within a universal service, and the patient failed to attend or failed to cancel in a timely fashion. The argument that missed appointments occur because patients forget, or they no longer need them, is common. It differentiates frivolous and irresponsible users of the health system from those with a ‘genuine’ need. The former fall short in their citizenship duty and collective responsibility to protect the NHS and help their fellow patients. Policy statements repeatedly return to need to “take personal responsibility”, ^16^ with the implication that missing an appointment is inherently irresponsible. Those missing *multiple* appointments are often subjected to particularly criminalising and stigmatising language, described as “repeat offenders” ^25^ or as lazy, shameless, disrespectful and entitled, emblematic of a wider breakdown in the social and moral order (e.g. ^26– 28^). This compounds when intersecting with other stigmatised statuses. Why should money be wasted on these patients, when it could be spent on those who more neatly fit into the deserving categories:

‘Each appointment costs an average of £30, putting the total cost to the NHS at more than £216 million pounds on top of the disruption for staff and fellow patients that would pay for:

• The annual salary of 2,325 full time GPs;
• 224,640 cataract operations;
• 58,320 hip replacement operations;
• 216,000 drug treatment courses for Alzheimer’s;
• The annual salary of 8,424 full time community nurses.’ ^18^

Further moral hierarchies are visible through the language of ‘genuineness’. There are ‘genuine’ or ‘valid’ reasons for missing appointments, often universal, logistical challenges (childcare, transport, work conflicts). ^1, 27, 29^ There are also patients whose ‘vulnerabilities’ are seen to limit their agency, and so their personal moral responsibility. ^1, 30^ Those whose vulnerabilities can be explained clinically are granted legitimacy; others are subjected to moral schema and their legitimacy denied. ^31, 32^ Moreover, while there is some recognition of the role of poverty or complexity in patients’ lives, ^33^ this is often framed in a way that brings a sense of intractability, situating these issues within the patient population, beyond the scope of any intervention to change. ^34^

### Intervention proposals

This morally evaluative, acausal approach influences the solutions proposed, which typically target patient behaviours and patient-side factors to produce resource savings for the service. Many are punitive, including removing patients from waiting lists or GP lists, or threatening to do so. Fines or charges are proposed; if a service is free, it is not valued, and payment may force reflection on responsibility. ^16, 35^ Even when rejecting fines, former UK Prime Minister David Cameron reaffirmed responsibility as the key issue:

‘When you have pressures on the NHS […] it’s important that we get people to take personal responsibility for the way that we use NHS resources.’ ^36^

**Figure 1.**
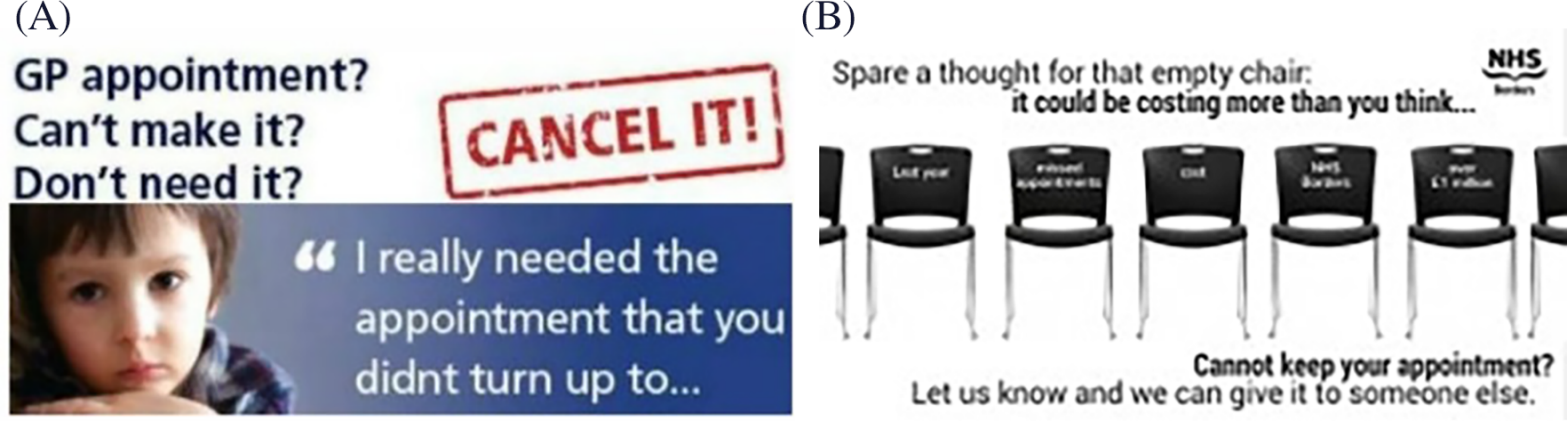
‘nudge’ examples from NHS services. ^1^

Fines were raised again by Rishi Sunak in his UK Conservative political party leadership campaign against Liz Truss, and they re-emerge regularly in public discourse. Less punitive interventions retain behavioural and normative components. Reminders address forgetfulness or disorganisation, encouraging attendance or timely cancellation. Behavioural science-informed approaches attempt to tweak or optimise patient behaviour by changing the ‘choice architecture’ around them. ^37, 38^ Patients are ‘nudged’ towards attendance by giving them information about the consequences of missed appointments for them, the wider community, or the NHS, or by communicating desirable and expected behavioural norms in reminders, letters, online posts, or waiting room posters ( Figure 1). ^38– 40^ Despite claims to scientific depth, legitimacy and objectivity, a normative focus on behavioural problems and individual responsibility is central to nudge approaches as they inherently contain a right and a wrong choice, the latter framed as an irrational error of judgement. ^41^ There are also concerns about impacts on those less able to act upon a nudge by virtue of their social and material circumstances, who risk being further pathologized as deeper and broader causes go unaddressed. ^42^

Even when addressing logistical concerns, interventions retain classical and normative components. Some allow patients to ‘manage’ their care through patient portals or apps. ^43, 44^ Language of management, convenience and choice preserves the classical model: patients remain responsible, while the health service facilitates this responsibility. Evening and weekend or telephone appointments are designed to address universal practical concerns. ^17, 18^ Other approaches do not even attempt to solve the problem, such as clinic overbooking – an approach that actively depends on patients not attending in order to maximise service efficiency. Among all of these, there are only occasional references to those vulnerable patients who need ‘particular consideration’, with little elaboration as to what that consideration might involve beyond protection from punitive practices, ^30^ and a need to explore and understand causes that the classical model has never actually established. ^30, 33^

### New developments: technological solutionism and the AI revolution

Recently, the problem of missed appointments has intersected with the wider narrative about the problem-solving potential of Artificial Intelligence (AI) in healthcare. ^17, 45, 46^ This aligns with the NHS 10-year plan, where growth in data availability and computing power are positioned as a route to efficiency, optimisation and resource savings. ^47^ Applying AI to vast quantities of data will transform care delivery and solve the classical problem by predicting missed appointments, allowing preventive or mitigatory action to protect the NHS and its resources:

“If you’re worried about waiting times - and aren’t we all - AI can save hundreds of thousands of hours lost to missed appointments because it can identify those on the list most likely not to turn up.” ^48^

Here, patients’ lives, circumstances, and experiences become quantifiable data, with the risk of a future missed appointment calculated by the algorithm which then makes decisions as to what should be done, and to whom. ^49^ Previously unknown patterns are uncovered as a function of the sheer volume of data, the algorithm’s complexity, or the alluring mystery of the secretive ‘black box’ ^50^ and its capacity to provide precise insights from disparate data …

‘… including people’s jobs, childcare commitments, live traffic and weather updates to determine who is most unlikely to show up and maximise doctors’ time.’ ^46^

There are strong signs of ‘technological solutionism’ ^51^ or ‘technochauvinism’ ^52^ here – the belief that social problems have technical solutions, and that the tech sector can succeed where governments have failed by bringing more objective insights without biases, politics or other human foibles. Here, social problems are matters of inefficiency, ‘bugs’ to be fixed with a combination of technology and an entrepreneurial mindset. ^53^ AI approaches often involve partnership with private companies who often provide both the prediction *and* the solution to a public sector stripped of its resources and capacities. ^53^ In the NHS 10-year plan, patient data will be given to AI businesses ‘to help them discover new breakthroughs and develop new products for health promotion and early detection’. ^47^

Yet this objectivity and promise of insight are illusory. Far from cutting through biases and other forms of noise, technology tends to embody them and to embed particular understandings of the world, restricting possibilities for action and acting as a vector for problematisations. ^54^ This is “algorithmic governmentality”, ^55^ existing biopolitical (or necropolitics) projects furthered by AI. Here, the goal remains the numerical reduction of missed appointments for the benefit of the service. The tools remain, but optimised. Clinics are overbooked; reminders targeted to high-risk patients at optimal times. ^17^ Crucially, this furthers the idea that a missed appointment is a single, discrete, predictable event with proximal causes and identifiable associations that can be used to optimise interventions. A pattern of missed appointments is relevant only as a sign of *risk* for a future missed appointment for the service. Enduring problems at a deeper level or on a broader scale, which are harder to quantify as data and may require broader political and structural changes, are understood only as they manifest at the individual level. ^56^ Poverty or ethnicity, for example, can be demographic risk markers for a missed appointment that require an optimised targeted response. *Addressing* poverty or racism more widely are never posited as a route to improved access, and the health and life experiences of ‘high risk’ groups are rarely part of the knowledge considered valuable in the predictive space. Predictive approaches can identify patients, but have little value unless critically integrated with other forms of knowledge and other approaches to the problem itself.

### A genealogy of the problem: missed appointment research

The classical problematisations enduring power reflects serious limitations in the research base. As our work highlights, ^4, 10^ research also reduces missed appointments to a quantitative problem – measuring rates, identifying correlations, calculating percentage reductions and cost savings. Causal insight comes from surveys often using pre-set, shallow, patient-side and behavioural components - “forgot”/“busy”/“competing demands” (i.e. were irresponsible or failed to manage their life) (e.g. ^38, 57, 58^); “symptoms resolved” (did not need the appointment and failed to cancel). ^59, 60^ There are no theoretical engagements beyond models of cognition, belief or behaviour. ^4^ We ‘know’ these issues cause waste, so reminders, nudges and booking systems become logical, evidence-based solutions. Measured on classical terms, many successfully reduce overall missed appointment rates. The knowledge base becomes a closed loop, its core findings regularly recreated through new correlation or survey studies in new settings, creating the illusion of an incontrovertible, objective evidence base.

Our work found that this research has silenced some patient voices through exclusionary recruitment and methods, and by ignoring the unequal uptake of interventions and/or distribution of their effects. ^4, 10^ Where the goal is the cost-effective reduction of missed appointments across whole services and quick wins can be achieved with minimal effort, there is no reason to explore more complex or ‘difficult’ patients. ^9^ A timely cancellation is valid because it reduces the non-attendance rate, regardless of whether the patient still needs care. A missed appointment in an overbooked clinic shows a successful predictive schedule. Very few studies explore whether attendance rate improvements come from patients who are deterred from making appointments. There are even fewer qualitative studies about multiple missed appointments generally. ^61^ This contributes to the lack of confidence and specificity in the classical problematisation around vulnerability or complexity, as studies often suggest ‘more intensive intervention’ ^62^ without elaboration beyond targeting the standard approaches at these groups.

Predictive research builds on these foundations, using future-oriented algorithms where prior work sought retrospective correlation. ^63^ Within the field, critics note low predictive accuracy when systems are trained and tested on the same data without external validation, ^64, 65^ or where they lack data on marginalised patient groups and thus recreate their marginalisation in the actions they take. ^56, 66^ Samorani et al. ^67^ describe how overbooking patients with a higher likelihood of missed appointments produced worse patient experience for Black patients because missed appointments and race were closely correlated. Other studies have similarly shown how attempts to optimise health provision using AI systems can embed exclusionary dynamics and result in marginalised groups receiving fewer resources – embedding, rather than resolving, inequities. ^68, 69^ To these critiques, we add our own – that predictive research is neither new nor revolutionary, instead embedding classical problematisations of health issues. Predictive papers continue to judge outcomes according to service-wide non-attendance rates and resource savings. Papers suggest familiar (optimised) solutions scheduling innovations or reminders, targeted and optimised by the predictive method. When these are less effective than hoped the predictive premise is not questioned. Answers are sought in different forms of analysis or different ways to manage variables or missing data ^63, 66, 67^; or in gathering more (and more personal) data. ^70, 71^ Yet consistently the variable most strongly correlated with future non-attendance is past non-attendance, ^62, 70, 72^ raising the question of whether complex predictive systems are necessary at all. Other strong associations include poverty and other forms of marginalisation, but these are again a site for identification and not intervention. The focus on specific, measurable, observable phenomena at the heart of predictive research discourages any emphasis on broader social circumstances and structures, or other forms of knowledge including the qualitative. ^56^ This monomaniacal pursuit of targeted optimisation is an extension of the problem articulated by Abraham Maslow:

“It is tempting, if the only tool you have is a hammer, to treat everything as if it were a nail.” ^73^

Extending this metaphor, the AI approach tends to *fashion nails in order to apply the force of the hammer in an optimal fashion,* without querying whether either hammer or nail is appropriate to the task.

### The missingness lens

How might we think differently, in terms of both practice and the evidence underpinning it? The first step involves addressing ‘structural missingness’, the epistemic violence that occurs where those excluded from healthcare are also excluded from knowledge production about it. ^74, 75^ It also requires addressing hermeneutic injustice, where the classical problematisation has dominated the interpretive space for so long that there is no alternative way for people to interpret and articulate missingness as a specific form of marginalisation. ^76^ Amplifying those voices and their alternative problematisations through inclusive, participatory and critical research is crucial, and is what we have aimed to do. When combining epidemiological work exploring the impacts of patterns of missed appointments *for patients*; interviews with seldom-heard patient groups; and a process of intervention co-design, the ‘problem’ looks quite different. This perspective can be summarised as a set of principles that we term the ‘missingness lens’, which has several core elements set in opposition to the classical model ( Table 1). ^10^

Rather than seeing missed appointments as an issue *for services* caused *by patients,* a missingness lens addresses barriers contributing to missingness *for patients.* The classical model frames appointment systems as equally accessible; the missingness lens see them as a poor fit for many patients, who are punished for failing to adhere to the system’s expectations. ^77^ For policymakers, this means reviewing incentive structures, targets, and service resourcing to meet the needs of ‘missing’ patients – not seeking cost-effective, service-wide reductions, but seeking to improve patient access and patient outcomes. Through proportionate universalism and positive selectivism, resources are put where they are most needed and targeted towards specific patient groups. ^78^ For services, this means meaningfully committing to identifying and addressing barriers leading to missingness. ^10^ One promising example is the Scottish Inclusion Health Action in Primary Care pilot, ^79^ which provides additional resources to practices, allowing them to proactively contact patients and to build a picture both of individual patients’ needs and collective barriers to care.

When patients are asked about what causes missingness, both deeper and broader causes are evident. Contributors were less focused on shallow behavioural and the practical issues and were more concerned with deeper ideas of *safety*, of mistrust and distrust, stigma and trauma, and the ways in which seeking care is a vulnerable act and health services are unsafe spaces for many patients. ^8, 9^ They prioritised changing interpersonal and institutional power dynamics, establishing trust, and addressing stigmatisation, retraumatisation and threat – all entirely absent in the classical model. Contributors also situated causes within social and structural conditions, deepening concepts like forgetting, logistics, vulnerability, competing demands. People are *vulnerabilised*, not inherently vulnerable; precaritised and exposed to multiple demands; denied access to the resources required for good health, good healthcare and other forms of security. ^9, 20^

Wider social policies and inequalities contribute through poverty, joblessness, homelessness, the erosion of the statutory safety net and of community resources. Many contributors were subject to inflexible demands from multiple systems - fragmented health services; punitive welfare systems; precarious employment arrangements – with some also managing these systems as carers. While occasional media pieces do explore system-level challenges beyond patients’ control – missed letters, short appointments, lack of local provision ^80– 83^ there is often little link to these wider *structural* inequities and the focus remains logistical. This is more than a practical issue – it becomes a source of psychological and emotional burden, linked to sense of self and to societal norms about how to manage one’s life. Consequently, contributors advocated for approaches that recognise and address how socioeconomic circumstances impact their health and healthcare encounters. ^9^ The missingness lens asks what interventions would look like if designed for (and by) those furthest from care, and how missingness might be addressed through actions aimed at these deeper and broader causes. While contributors did suggest some ‘classical’ solutions - reminders, convenience, transport - they did so from a fundamentally different orientation where the needs of ‘missing’ patients were central to the design and implementation of these interventions – not an ‘additional consideration.’

## Conclusion

“The problem, to be quite honest with you, is that you’ve never actually known what the question is. […] Once you do know what the question actually is, you’ll know what the answer means.” ^84^

Applying a missingness lens does not mean we entirely dispose of the existing corpus of knowledge, nor with the approaches or interventions detailed above. We need instead to address their limitations, and to embed them in roots that allow them to flourish. Identifying patients at risk of missingness is important, and scrutiny of patient records is central to this. ^10^ But they are the precursor, not the answer. Where ‘DNA rates’ are measurable, discrete entities, missingness is a proxy for enduring access issues or wider adversities. As the supercomputer Deep Mind suggests in the *Hitchhiker’s Guide to the Galaxy* above *,* we need to carefully consider the questions we ask of technology, or we risk fundamentally misinterpreting the nature of the world around us. Addressing missingness requires an understanding of patient outcomes beyond simply attendance; of understanding and addressing deep causes of missingness at individual, relational, institutional and structural levels; and of the ways in which interventions can either exacerbate or mitigate access inequalities. Encouraging work in the predictive space suggests using stakeholder input to set research questions, outcomes, and variables, or complements predictive insights with qualitative insights from high-risk groups. ^50, 85^

For researchers and funders, there is a need to address epistemic violence and injustice by moving away from correlative and survey studies towards qualitative, participatory research with people from marginalised patient groups; stronger and more critical theoretical engagements; and intervention studies that account for missingness in sampling and outcome reporting. There is also a need for more critical evidence syntheses. Missingness insights can be found in work exploring healthcare experiences for marginalised patient group (e.g. inclusion health, HIV care, minoritised communities. ^86– 90^ These papers very rarely appear in systematic reviews, which rely so often on the binary, pre-set outcome of “non-attendance rates”, and on the limited and restrictive methodologies above. A form of methodological segregation has emerged: the mainstream of health service research and the peripheral Others whose knowledge is limited to ‘niche’ services. This needs to be addressed.

Ultimately, our hope is that missingness becomes a way to address health and access inequalities, but also to address these epistemic injustices. Along with empirical insights, our work provides a conceptual language to make sense of a cross-cutting form of marginalisation and a schema to resist stigmatising and exclusionary approaches in research, policy and practice. Applying a missingness lens is a guiding principle for targeting interventions towards patients in a way which does not position them as problematic, but which instead problematises the wider contexts within which healthcare inequalities emerge. Researchers, policy makers and practitioners should instead as their starting point think - what do we need to do differently to include all patients in care?

## Ethics and consent statement

The College of Medical, Veterinary and Life Sciences Research Ethics Committee of the University of Glasgow gave ethical approval for this work (Reference: 200220187).

Written informed consent for publication and participant obtained from patients’ and their images was obtained from the participants/patients/parents/guardian/relative of the participant/patient.

## Award information

National Institute for Health Research (NIHR), UK [study identification 135034].

## Data Availability

Underlying and extended data: 55 interview transcripts can be accessed on reasonable request via an email to the corresponding author or to shw-missingness@glasgow.ac.uk. The following conditions apply: new users will not attempt to de-identify research participants, new users will emulate the values of the original research purpose and not seek to stigmatise individuals or communities, and no UK or international laws will be broken. Due to the sensitive disclosive nature of some of the transcripts 5 are closed to access. One interview was not transcribed. The University of Glasgow MVLS Ethic Committee and University of Glasgow Research Data Management Team approved this approach. Data relating to review methods can be found in papers relevant to those reviews. ^4, 10^ The full list of news articles is available within the University of Glasgow Enlighten repository and is available freely: DOI: 10.5525/gla.researchdata.2280. Data are available under the terms of the Creative Commons Attribution 4.0 International license (CC-BY 4.0).
