# Supplementary material for: What are we missing? Reflections on the ‘problem’ of missed appointments in the UK
Source: NIHR Open Res. 2026 May 19;6:23. Originally published 2026 Mar 10. [Version 2] doi: 10.3310/nihropenres.14239.2 (PMC13247576; doi:10.3310/nihropenres.14239.2)
Supplement: Peer review history

# Reviewer report: Reviewer response for version 2

**DOI:** 10.3310/nihropenres.15617.r40544

**Published:** 2026-06-16

**Authors:** Margarita Corry

**Affiliations:** 1 Trinity College Dublin, Dublin, Ireland

This paper is a critical commentary on findings from a larger project, which is either indexed in peer review journals or publicly available and referenced within the paper.  The paper highlights an important concern in relation to the use of AI to predict missingness and how this is limited by the dominant discourse. The paper is timely and the discussion on the limitations of using AI to inform policy and practice is of value beyond the topic explored in this paper.

 The focus of the paper is clearly articulated and consistent across the abstract, plain language summary and main body of the paper.  

 The background outlines the problem with missingness and the impact of this. The problem with current discourse and the argument for the need for a different approach is clearly articulated.  The framework used for policy analysis is presented and how it was applied across the different sources of data explained.

**The focus of the paper is clearly presented.**

 On page 6 the heading ‘Results’ is a little confusing as this paper is not reporting on a primary study.  It is a critical commentary on the models of missingness which were developed based on the findings of the project. **I recommend that the authors consider the accuracy of the heading ‘Results’.**

 Page 6/18 states:

 “Health policy problematizations define who subjects are, what they might become, and how they should be governed bio politically– through the regulation, administration and management of their lives and health, including through healthcare.14 Necropolitical governmentality administers not life but death, determining who is worthy of care and who is “disposable”,19 whose suffering is permissible and less worthy of attention because of their position in a moral hierarchy. 20 Mirroring wider discourse in health policy,21,22 statements about waste and lost capacity divide the patient population into those who are worthy of resources (and who pay for them through taxes), and those who are unworthy, whose difficulties are a function of their own shortcomings, and who drain resources from society.20,23”

**The example provided for that worthy of resources is distracting and not evidenced.  If references 20 or 23 directly supports that assertion, then I recommend the reference is also placed after the example. If not, I recommend that the example is removed or replaced by one that can be evidenced.**

 Pathologized spelling UK English has an s not z.

 Other than these minor issues I believe the argument presented in this paper is well developed and clearly articulated.  The project which this paper reports on along with evidence from the wider literature support the arguments presented.

Is the work clearly and accurately presented and does it cite the current literature?

Yes

If applicable, is the statistical analysis and its interpretation appropriate?

Not applicable

Are all the source data underlying the results available to ensure full reproducibility?

Partly

Is the study design appropriate and is the work technically sound?

Yes

Are the conclusions drawn adequately supported by the results?

Yes

Are sufficient details of methods and analysis provided to allow replication by others?

Yes

Reviewer Expertise:

Complex intervention development, Survey, Mixed Methods, Randomised Controlled Trial design and qualitative methodologies.

I confirm that I have read this submission and believe that I have an appropriate level of expertise to confirm that it is of an acceptable scientific standard.

# Reviewer report: Reviewer response for version 2

**DOI:** 10.3310/nihropenres.15617.r40548

**Published:** 2026-06-16

**Authors:** Louise Laverty

**Affiliations:** 1 University of Leeds, Leeds, UK

This paper presents a compelling case for unpicking mainstream narratives around missed appointments in the UK, pulling together the evidence to highlight how these narratives are reinforced and embedded into healthcare organizations and practices. 

 The paper is well written, well-evidenced, and provides a coherent journey for readers to understand the issues in depth, alongside presenting a proposed solution. All too often, policy analysis of this type helps demonstrate the problem without offering ways to address it. 

 I have two recommendations for the authors.

 Firstly, I think the authors may confuse readers with their positioning of the paper as both a commentary piece and a policy analysis and sell short the value of the work. I would suggest simply calling it a critical policy analysis (as I would assume most policy analysis would have a critical commentary of some sort to give it context). 

 My second recommendation would be to add more detail about the analysis process for the policy review. I appreciate the use of Bacchi to guide the analysis, but there is less information for the reader about how the documentary material was managed, coded, sorted, and organized to guide their results and how this differed by applying Bacchi's questions. The level of detail is not uncommon in policy reviews, but it would be a strength to provide greater depth to the process.

Is the work clearly and accurately presented and does it cite the current literature?

Yes

If applicable, is the statistical analysis and its interpretation appropriate?

Not applicable

Are all the source data underlying the results available to ensure full reproducibility?

Yes

Is the study design appropriate and is the work technically sound?

Yes

Are the conclusions drawn adequately supported by the results?

Yes

Are sufficient details of methods and analysis provided to allow replication by others?

Partly

Reviewer Expertise:

Medical sociology, health services research, inequalities in healthcare access.

I confirm that I have read this submission and believe that I have an appropriate level of expertise to confirm that it is of an acceptable scientific standard.

# Reviewer report: Reviewer response for version 2

**DOI:** 10.3310/nihropenres.15617.r40542

**Published:** 2026-06-15

**Authors:** Daria Morgounova Schwalbe

**Affiliations:** 1 Odense University Hospital and University of Southern Denmark, Odense, Denmark

**Summary**

 This article offers a critical perspective on dominant approaches to missed healthcare appointments, making a compelling contribution to the field. Drawing on post-structuralist theory, particularly Carol Bacchi’s analytical approach, which uses the concept of problematization to direct attention to the heterogeneous strategic relations and politics that shape lives, the paper examines the disparity between how missed appointments are represented and governed and users' perspectives on missed appointments. The article situates missed appointments within contemporary crisis rhetoric and forms of necropolitical governmentality, which I found both novel and thought-provoking. Drawing on insights from interviews and workshops conducted as part of the Missingness project, alongside a review of research literature and news materials, the paper highlights important tensions between participants’ experiences and dominant policy and media representations. It argues that much existing research reduces missed appointments to a quantitative and managerial problem, thereby silencing patient voices and obscuring structural inequalities and forms of epistemic injustice, and advocates for an alternative way of thinking about missed appointments – through a “missingness lens” that sees missed appointments not primarily as a problem caused by patients for healthcare services, but as an indicator of barriers to care access experienced by patients.

 A central contribution of the paper is its critique of current research and intervention strategies, including the increasing reliance on AI and predictive methodologies. The author argues that the promise of insight offered by AI is, to some extent, illusory. It reinforces the assumption that missed appointments are discrete, predictable events with identifiable proximal causes that can be managed through optimization and redirects attention away from broader structural issues such as poverty, racism, exclusion, and epistemic injustice. The article argues convincingly that such approaches risk reproducing exclusionary dynamics and further marginalizing already disadvantaged groups. The argument that people are vulnerabilized rather than inherently vulnerable is also an important and valuable. The article claims to provide a conceptual language for understanding cross-cutting forms of marginalization.

 The conclusion summarizes the implications of applying a missingness lens and advocates for greater stakeholder involvement and qualitative and participatory approaches to complement quantitative and predictive forms of research.

**Overall Evaluation**

 Overall, I found this article highly engaging, original, and important. The argument is compelling, clearly articulated, and well supported by relevant literature. The framing of missed appointments within crisis rhetoric and necropolitical governmentality is innovative and compelling. I particularly like the shift from what the author terms the “classical model,” in which missed appointments are framed as deviant behavior and patient responsibility, towards a missingness lens that foregrounds structural barriers, silencing, and exclusion. The discussion of how forms of governmentality produce distinctions between those deemed worthy of resources and those deemed unworthy is especially thought-provoking. The study design is appropriate for the article’s aims, and the critical perspective offered is both timely and much needed.

 The work cites a vast amount of relevant literature. I suggest including a recent study on non-attendance in Danish healthcare, which supports several of the article’s core arguments regarding access barriers, structural exclusion, and emotional burden. 

**Comments and suggestions**

**1. Method**

 Although the article is primarily a conceptual and critical analysis rather than a presentation of original qualitative findings, the Missingness project appears to play an important role in informing the argument. For this reason, more detail is needed in the method section. The manuscript states that realist interviews were conducted with 61 individuals with personal and professional experience of missed appointments. However, it remains unclear who these participants were. Were they patients, relatives, healthcare professionals, policy stakeholders, or a combination of these groups? A brief table describing participant characteristics would be helpful.

 In addition, I would encourage the author to clarify:

- Whether interviews and workshops were audio-recorded and transcribed;
- What forms of documentation were used;
- How the material was analyzed;
- How insights from these data informed the development of the missingness lens.

Providing this information would strengthen the transparency and credibility of the paper and support the core argument for the missingness lens framework.

**2. The presentation of data sources**

 The distinction between “Initial data collection” (the Missingness project) and “Additional data collection” (policy and news materials) feels somewhat unusual. Given the nature of the article, it may be methodologically better to present the sources on equal terms as:

1. Review of academic and grey literature;
2. Interviews and participatory workshops;
3. Review of news materials.

This is a relatively minor point, but I believe it would improve methodological coherence, particularly since most of the discussion in the article (before presentation of the missingness lens) is built primarily on a critical review of literature and news materials.

**2. The relationship between empirical material and analytical claims**

 Several important claims appear to be derived from the Missingness project, but it is not always clear whether they emerge from participant accounts, represent analytical interpretations, or are based on existing literature. For example, statement: “ *People are vulnerabilised, not inherently vulnerable.*” While I find this argument persuasive, there is a substantial theoretical literature debating vulnerability. It would therefore be helpful if the author clarified whether this conclusion emerged from the empirical material, how it was reflected in participants’ accounts, and how it relates to existing scholarship.

 Similarly, statements such as: “ *Many contributors were subject to inflexible demands from multiple systems…” *and references to psychological and emotional burdens would benefit from clearer empirical grounding. If these observations emerged from interviews or workshops, I encourage the author to provide illustrative examples. If they represent analytical conclusions, the manuscript should explain how they are supported by the findings.

 More generally, the section introducing the missingness lens would benefit from:

1. Explicitly stating from the outset that it builds on insights from interviews and participatory workshops;
2. Briefly presenting the main findings – themes or assertions emerging from these data;
3. Supporting the discussion with a small number of empirical examples that help distinguish participant perspectives from the author’s analytical interpretation.

**4. Define key concepts more clearly**

 The discussion of necropolitical governmentality, algorithmic governmentality, epistemic violence, hermeneutic injustice, and exclusionary language is interesting and relevant. However, some concepts would benefit from further explanation. For instance, the concept of necropolitical governmentality is central to the manuscript's argument, yet it remains somewhat underdeveloped. I encourage the author to provide a more explicit definition of the concept and – if not clarify how it extends or differs from Foucault's notion of governmentality – at least explain how necropolitical governmentality operates in the context of missed appointments and how it relates to neoliberal healthcare logics, personal responsibility, self-management, and the allocation of care resources. For someone unfamiliar with the concept, the argument presented on p. 6 is difficult to follow.

 Similarly, the manuscript refers to: “the directly exclusionary language of (ir)responsibility, entitlement, chaos and non-compliance” but does not provide concrete examples. Including examples from the empirical material would strengthen this argument considerably. Schwalbe et al. (2026), for example, provide illustrations of similar forms of language use in healthcare contexts.

**5. Wording and consistency in terminology**

 In the section “Genealogy of the problem,” the manuscript repeatedly refers to “research” as reducing missed appointments to a quantitative problem. I suspect the author is primarily referring to epidemiological, biomedical, health services, or predictive modelling research rather than research more broadly. Clarifying this would make the critique more precise and avoid overgeneralization.

 Similarly, on page 9, the statement: “Our work found that this research has silenced some patient voices through exclusionary recruitment and methods…” would benefit from greater specificity. It may be more accurate to refer to particular methodologies or research traditions rather than “research” in general. It would also be useful to clarify who these “some patients” are and whether specific groups are disproportionately excluded.

 Throughout the manuscript, terms such as “people,” “patients,” and “contributors” are sometimes used interchangeably. This occasionally makes it difficult to determine whether contributors refer only to patients or also include healthcare professionals and other stakeholders. Clarifying participant categories in the methods section and using terminology more consistently throughout the manuscript would improve readability.

**6. Conclusion**

 Finally, I would encourage the author to return more explicitly to the six problematization questions introduced on page 5 when developing the conclusion. These questions are important and provide the analytical foundation of the paper and revisiting them would help integrate the findings and discussion more closely with Bacchi’s framework.

 The manuscript concludes by arguing that researchers, policymakers, and practitioners should ask: *“What do we need to do differently to include all patients in care?”* This is an important argument. I would encourage the author to extend this reflection further by suggesting that researchers, policymakers, and practitioners might start by critically examining their own assumptions, practices, and forms of knowledge production, including the potential consequences of increasingly digitalized and AI-driven approaches to healthcare governance.

**Conclusion**

 I definitely recommend the article for indexing. The article is insightful, thought-provoking, and it makes an important contribution to ongoing debates about missed appointments, healthcare access, and structural exclusion. The argument is original, persuasive, and timely. The revisions are minor and concern methodological transparency and greater clarity regarding the relationship between empirical material and analytical claims. Addressing these issues would significantly strengthen the manuscript while preserving its innovative conceptual contribution

Is the work clearly and accurately presented and does it cite the current literature?

Yes

If applicable, is the statistical analysis and its interpretation appropriate?

Not applicable

Are all the source data underlying the results available to ensure full reproducibility?

Yes

Is the study design appropriate and is the work technically sound?

Yes

Are the conclusions drawn adequately supported by the results?

Partly

Are sufficient details of methods and analysis provided to allow replication by others?

Partly

Reviewer Expertise:

health communication, neoliberal care logic, nonattendance, hospital-initiated cancellations, mental health, exclusion, inequality, marginalisation, silencing,  PAR, co-creation, user perspectives, ethnography, conceptual metaphor framework, interactional dynamics

I confirm that I have read this submission and believe that I have an appropriate level of expertise to confirm that it is of an acceptable scientific standard.

## References

[1] : Absent or not invited? A multilevel qualitative study of patients and healthcare professionals’ perspectives on barriers and enablers of outpatient attendance in Southern Denmark. *BMC Health Services Research* .2026; 10.1186/s12913-026-14533-y 10.1186/s12913-026-14533-y PMC1327079442045889

# Reviewer report: Reviewer response for version 2

**DOI:** 10.3310/nihropenres.15617.r40545

**Published:** 2026-06-08

**Authors:** Matthew A Jay

**Affiliations:** 1 University College London, London, England, UK

This is a very interesting study and a very welcome addition to the literature. Although not an aim of this study, a similar lens on rejected referrals and other similar access issues would be interesting (indeed, not attending may be a reason for rejecting a referral without seeing a patient so these issues are clearly very closely tied together and likely share common cause). Perhaps some discussion on these issues as questions for further development/research could be addressed in the paper. I also wonder to what extent these issues share common cause with data showing longer waiting times for NHS treatments for certain groups.

 I am reviewing version 2 of this paper and can see that the authors have already addressed a number of comments related to the clarity of the methods, such as including search terms. However, I do feel the paper is still somewhat difficult to follow at times as, although an empirical paper, it is not presented in quite the same way as an empirical paper. I appreciate the authors are explicitly presenting this is "part research paper, part critical commentary" but this does I feel tend to obscure some of the methodological detail, making it harder to get one's head around the "research paper" part of it.

 In particular, it is not immediately clear where the classical model/missingness lens table comes from. It seems that this is fruit of the study and should really be in the results section, and it should be clearer how this was developed. Or perhaps it was developed a priori, but this needs to be clearer. The results section is then similarly somewhat difficult to follow as it is not always clear whether what is presented is a result of the literature review part, the interview part, the workshop part or the authors' own interpretation when writing the manuscript. Indeed, to be really brutally honest, I am not sure what in the manuscript comes from the interviews and workshops as results from those are not obviously presented.

 In sum, I think this is a valuable contribution but my personal view is that the manuscript could do with some reworking to draw out the empirical parts more, leaving space in a discussion section for the critical commentary

Is the work clearly and accurately presented and does it cite the current literature?

Partly

If applicable, is the statistical analysis and its interpretation appropriate?

Not applicable

Are all the source data underlying the results available to ensure full reproducibility?

Yes

Is the study design appropriate and is the work technically sound?

Yes

Are the conclusions drawn adequately supported by the results?

Yes

Are sufficient details of methods and analysis provided to allow replication by others?

Partly

Reviewer Expertise:

Health and social care research; health inequailities; access to health care

I confirm that I have read this submission and believe that I have an appropriate level of expertise to confirm that it is of an acceptable scientific standard, however I have significant reservations, as outlined above.

# Reviewer report: Reviewer response for version 2

**DOI:** 10.3310/nihropenres.15617.r40516

**Published:** 2026-05-23

**Authors:** Emma Parry

**Affiliations:** 1 Keele University, Keele, England, UK

Thank you for addressing my comments.

Is the work clearly and accurately presented and does it cite the current literature?

Partly

If applicable, is the statistical analysis and its interpretation appropriate?

Not applicable

Are all the source data underlying the results available to ensure full reproducibility?

Partly

Is the study design appropriate and is the work technically sound?

Partly

Are the conclusions drawn adequately supported by the results?

Yes

Are sufficient details of methods and analysis provided to allow replication by others?

No

Reviewer Expertise:

Primary care, mixed methods

I confirm that I have read this submission and believe that I have an appropriate level of expertise to confirm that it is of an acceptable scientific standard.

# Reviewer report: Reviewer response for version 1

**DOI:** 10.3310/nihropenres.15504.r40084

**Published:** 2026-04-16

**Authors:** Sahar Yazdanfard

**Affiliations:** 1 University of Houston, Houston, Texas, USA

**Plain Language Summary**

 Could you clarify what is meant by  ‘saving even more money’ in this sentence,  “This approach is now starting to influence Artificial Intelligence-based interventions that promise to predict who will miss appointments, saving even more money.”

**Method**

**C**heck the readability of the sentence, ' In the conceptualization and development phase, members of the research team consulted the Royal College of GPs Scotland Patient Participation in Practice (P3) and carried out pilot interviews, including with people with lived experience of missingness.'

 Provide more description of what these perspectives are: 'Throughout the study, we came across news articles, press releases, websites, even direct communications from our own GPs, that stood in direct contradiction to the good practice suggested by interviewees, stakeholders, and the rare pieces of research that applied a different perspective (e.g. ^11, 12^).'

 Can the authors explain more about how the method iterations were changed? Was there a tracker?

**Conclusion**

 "For researchers and funders, there is a need to address epistemic violence and injustice by moving away from correlative and survey studies towards qualitative, participatory research with people from marginalized patient groups; stronger and more critical theoretical engagements; and intervention studies that account for missingness in sampling and outcome reporting. " The authors' conclusion appears not to be supported throughout the article. Please consider addressing this.

 Authors need to provide references to support their concluding remarks, flagging to the reader that these ideas were not thoroughly explained throughout the body of the paper. Please consider making changes.

Is the work clearly and accurately presented and does it cite the current literature?

Yes

If applicable, is the statistical analysis and its interpretation appropriate?

Not applicable

Are all the source data underlying the results available to ensure full reproducibility?

Yes

Is the study design appropriate and is the work technically sound?

Partly

Are the conclusions drawn adequately supported by the results?

Partly

Are sufficient details of methods and analysis provided to allow replication by others?

Partly

Reviewer Expertise:

Quality Assurance;

I confirm that I have read this submission and believe that I have an appropriate level of expertise to confirm that it is of an acceptable scientific standard, however I have significant reservations, as outlined above.

# Reviewer report: Reviewer response for version 1

**DOI:** 10.3310/nihropenres.15504.r39967

**Published:** 2026-03-31

**Authors:** Emma Parry

**Affiliations:** 1 Keele University, Keele, England, UK

Thank you for inviting me to review this article, which presents interesting and relevant findings on missed appointments within the NHS. Overall, I found the methods section difficult to follow (see points below), and the introduction would benefit from more effectively setting the context—specifically by outlining the scale of the problem, summarising relevant prior research, and clearly identifying the gaps this study aims to address. That said, the findings, critical reflection, and conclusions offer a valuable contribution to this important area.

**Grammar, spelling:**

 Abstract/results “that seek alter” to “that seek to alter”

 his sentence needs updating “In a time of scarcity appointments are previous and valuable commodities in a way they may not have been before and wasting them is particularly egregious.”

 Update sentence: “How they are addressed has consequences for people’s health.”

**Introduction:**

 It would help readers if the sections that details the problem of missingness are moved to the introduction. I think the article would read better if the summary of the problem of missingness is described here, including scale of missed appointments, costs, where in the health system missingness is a problem (e.g. is it a bigger problem in primary or secondary care), cost and individuals who are more likely to “miss” appointments.

 It would be helpful to understand more about the findings of the work the authors previously published on causes of missingness and the further questions it raised. Authors could expand more on how this study follows on from that and addresses gaps.

 The aim of this paper is not very clear from the introduction and this should be specifically stated.

**Methods:**

 I found the methods confusing and difficult to follow, this limits the reproducibility of this work. I think adding subheadings would help in guiding the reader.

 The authors state in the abstract they will provide a critical commentary on findings from a realist review, key informant interviews and Stakeholder workshops. I think this could be stated more clearly in the methods that it is the data or findings from these studies that are being critically synthesised.

 The 3 data sources for this article could be more clearly summarised/defined in the methods.

 I’m not sure the “classical model” is in the right place. There is no context to the model where it is currently situated in the text-I think moving the description of the model before the model may help readability. It is not clear if the authors are wanting to present this as a “result”.  It also does not have a citation.

 Finally it is not clear how the search for news media was undertaken? Further details about this would be helpful.

**Results**

 There isn’t a results section so it was difficult to understand where the authors transition from describing methods to presenting results.

Is the work clearly and accurately presented and does it cite the current literature?

Partly

If applicable, is the statistical analysis and its interpretation appropriate?

Not applicable

Are all the source data underlying the results available to ensure full reproducibility?

Partly

Is the study design appropriate and is the work technically sound?

Partly

Are the conclusions drawn adequately supported by the results?

Yes

Are sufficient details of methods and analysis provided to allow replication by others?

No

Reviewer Expertise:

Primary care, mixed methods

I confirm that I have read this submission and believe that I have an appropriate level of expertise to confirm that it is of an acceptable scientific standard, however I have significant reservations, as outlined above.
